# Evaluation of FVIII pharmacokinetic profiles in Korean hemophilia A patients assessed with myPKFiT: a retrospective chart review

**DOI:** 10.1007/s44313-024-00023-9

**Published:** 2024-08-28

**Authors:** Young-Shil Park, Ki-Young Yoo, Sang Kyu Park, Taiju Hwang, Aeran Jung, Eun Jin Choi

**Affiliations:** 1 Department of Pediatrics, Kyung Hee University Hospital, Gangdong, Seoul, Republic of Korea; 2 Korea Hemophilia Foundation Clinic, Seoul, Republic of Korea; 3 Korea Hemophilia Foundation Clinic, Busan, Republic of Korea; 4 Korea Hemophilia Foundation Clinic, Gwangju, Republic of Korea; 5 Medical Affairs, Takeda Pharmaceuticals Korea Co., Ltd, Seoul, Republic of Korea; 6 Department of Pediatrics, Daegu Catholic University Medical Center, 33 Duryugongwon-Ro 17-Gil, Nam-Gu, Daegu, 42472 Republic of Korea

**Keywords:** Hemophilia A, FVIII, Pharmacokinetics, Prophylaxis, Korean

## Abstract

**Purpose:**

This study aimed to investigate the pharmacokinetics (PK) of factor VIII (FVIII) in Korean patients, as limited information is available on the PK of FVIII in this population.

**Methods:**

We collected the FVIII PK results from patients with moderate-to-severe hemophilia A using myPKFiT. PK variations were assessed according to age, blood type, inhibitor history, von Willebrand factor antigen (vWF:Ag) level, and body mass index. Additionally, the correlation between the PK profile and prophylaxis regimen was specifically analyzed for each product in severe cases.

**Results:**

The PK data of 48 and 81 patients treated with octocog alfa and rurioctocog alfa pegol, respectively, were obtained. The median half-lives of octocog alfa and rurioctocog alfa pegol were 9.9 (range: 6.3–15.2) h and 15.3 (range: 10.4–23.9) h, respectively. The PK profiles for each product did not differ according to age group; however, blood type-O patients had shorter half-lives and time to 1% compared to non-blood type-O patients. In regression analysis, the PK of octocog alfa showed a statistically significant difference according to age, whereas the PK of rurioctocog alfa pegol correlated with vWF:Ag. Only the frequency of rurioctocog alfa pegol use showed a statistically significant difference in relation to time to 1%, although the coefficient of determination was small.

**Conclusion:**

This study confirmed significant interpatient variation in the PK of FVIII among Korean patients with hemophilia A. To achieve optimized prophylaxis, personalizing the regimen based on the PK profile of each individual patient is essential.

## Introduction

The standard of care for individuals with severe hemophilia, as well as for some moderate hemophilia patients with a severe bleeding phenotype, involves prophylaxis, which is a regular replacement therapy designed to prevent bleeding and mitigate long-term consequences [1]. Effective prevention and treatment of bleeding in hemophilia requires plasma factor activity to exceed a defined target level. However, considerable interpatient variability in the pharmacokinetics of factor concentrates suggests that relying on the average pharmacokinetic (PK) characteristics of such concentrates would not enable optimal treatment for individual patients [2]. Additionally, this substantial interpatient variation in PK results in significant fluctuations in the required factor VIII (FVIII) dose to sustain the desired trough level for prophylaxis [3].

Guidelines for pharmacokinetic studies involving FVIII concentrates recommend 10–11 blood samples over a period of 32–48 h (additional samples extending to 96 h or longer for the extended half-life of FVIII) [1]. However, for dose tailoring in routine practice, useful PK parameters can be estimated using a smaller number of blood samples by leveraging population PK models using the Bayesian method [4]. One notable tool is myPKFiT, a web-based device that employs the Bayesian model to estimate the PK curve of octocog alfa and rurioctocog alfa pegol (ADVATE®; ADYNOVATE®; Baxalta US Inc., a Takeda company, Lexington, MA, USA). The recommended times for blood sampling for PK simulation using myPKFiT, as stated in the myPKFiT Healthcare Professional User Manual, are first at 3−4 h (± 30 min) after infusion for both products, and the second at 24−32 h (± 1 h) for octocog alfa and at 48 h (± 2 h) and/or 72 h (± 2 h) for rurioctocog alfa pegol [5]. This tool helps tailor prophylactic regimens for each patient [6] and was approved by the Korean Ministry of Food and Drug Safety. This medical device is the most widely used population PK tool for patients with hemophilia A in South Korea.

In Korea, restrictions on the reimbursement of factor concentrates pose a challenge to the implementation of personalized treatments for patients with hemophilia [7]. Moreover, Korean data regarding the PK of FVIII and its interpatient variability is limited. Understanding the PK profile of FVIII would be imperative for optimizing hemophilia treatment outcomes. Therefore, this study aimed to analyze the real-world PK of FVIII in Korean patients with hemophilia A.

## Methods

### Study design, patients, and data collection

A retrospective chart review was conducted across five Korean hemophilia treatment centers located in Seoul, Daegu, Busan, and Gwangju. FVIII PK data were collected using myPKFiT between January 1, 2018, and November 30, 2021. We included patients with moderate to severe hemophilia A (baseline FVIII < 5 IU/dL) within the age range of 1–65 years for octocog alfa and 12–65 years for rurioctocog alfa pegol, who had PK test results at the commencement of the study. Patients with detectable levels of FVIII inhibitors (antibodies) at the time of PK testing, those diagnosed with congenital or acquired hemostatic disorders other than hemophilia A, and those enrolled in an interventional hemostatic trial during the PK testing period were excluded.

The primary objective was to describe the FVIII PK of octocog alfa and rurioctocog alfa pegol in Korean patients with hemophilia A. Differences in the PK profiles (half-life, time to 1%, and clearance) of octocog alfa and rurioctocog alfa pegol, controlled for age, blood type, inhibitor history, von Willebrand factor antigen (vWF:Ag) level, and body mass index (BMI) were also analyzed.

### Statistical analysis

Data are presented as median (minimum, maximum), mean ± standard deviation (SD), or n (%). Group differences for each product were compared using parametric and non-parametric tests, as deemed appropriate. The Shapiro–Wilk test was applied to verify a normal distribution, and parametric tests were used to compare variables with a normal distribution. Parametric (independent t-test and ANOVA) or non-parametric (Mann–Whitney and Wilcoxon) methods were used for the analysis. BMI was derived from height and weight and categorized according to Korean guidelines. The association between the PK profile (half-life, time to 1%, and clearance) and age, BMI, and vWF:Ag was analyzed using a simple linear regression model. Age (< 6 years, ≥ 6– < 12 years, ≥ 12– < 18 years, and ≥ 18 years), blood type (O and non-O), and BMI (< 18.5 kg/m^2^, 18.5–22.9 kg/m^2^, 23.0–24.9 kg/m^2^, and ≥ 25.0 kg/m^2^) were treated as categorical variables.

Statistical significance was set at 5% (*P* < 0.05). Statistical analyses were performed using SAS version 9.4 (SAS Institute Inc., Cary, NC, USA) and R version 4.2.2 (R Foundation, Vienna, Austria).

### Ethics statement

The study protocol and the associated case report forms (CRFs), were reviewed and approved by the Public Institutional Bioethics Committee of the Ministry of Health and Welfare (approval no. P01-202206–01-021).

## Results

### Demographic and baseline characteristics

A total of 129 patients with moderate to severe hemophilia A and available PK data were identified. Among them, 48 and 81 patients were analyzed for octocog alfa and rurioctocog alfa pegol, respectively. 

Forty-seven patients (97.9%) in the octocog alfa group and all patients (100%, *n* = 81) in the rurioctocog alfa pegol group were male. The age range of patients using octocog alfa was 5–52 years, with a mean age of 23.21 years, whereas that of those using rurioctocog alfa pegol was 14–64 years, with a mean age of 27.60 years. Forty-one patients (85.4%) using octocog alfa and 70 patients (86.4%) using rurioctocog alfa pegol were classified as severe cases, characterized by a baseline FVIII level < 1% (Table 1).

**Table 1:** Demographic data

Variables	Octocog alfa (*N* = 48)	Rurioctocog alfa pegol (*N* = 81)	*P-*value
	Mean ± SD or N (%)	Mean ± SD or N (%)	
Sex			
Male	47(97.92)	81(100.00)	0.3721
Female	1(2.08)	-	
Age, years	23.21 ± 10.66	27.60 ± 10.04	0.0779
Height, cm	162.60 ± 23.09	172.66 ± 5.23	0.0898
Weight, kg	59.64 ± 23.36	73.30 ± 12.47	**0.0035**
BMI, kg/m^2^	23.29 ± 4.27	24.78 ± 3.78	0.0545
< 18.5	7(14.58)	2(2.47)	**0.0369**
18.5–22.9	13(27.08)	21(25.93)	
23.0–24.9	5(10.42)	18(22.22)	
≥ 25.0	17(35.42)	31(38.27)	
Blood type			
A	15(31.25)	27(33.33)	0.2370
B	8(16.67)	21(25.93)	
AB	3(6.25)	5(6.17)	
O	15(31.25)	12(14.81)	
Unknown	7(14.58)	16(19.75)	
FVIII level (Baseline)			
Severe (< 1 IU/dL)	41(85.42)	70(86.42)	0.8737
Moderate (≥ 1– < 5 IU/dL)	7(14.58)	11(13.58)	

*BMI* Body mass index, *SD* Standard deviation

Six patients (12.5%) in the octocog alfa group and 5 patients (6.2%) in the rurioctocog alfa pegol group had history of FVIII inhibitors. In each group, 5 patients received immune tolerance induction (ITI) treatment, and in all cases, the results were categorized as “complete response.” The prevailing FVIII treatment regimen at the time of the PK test was ‘prophylaxis,’ with a mean dose of 29.1 ± 4.7 IU/kg for the octocog alfa group and 28.8 ± 4.0 IU/kg for the rurioctocog alfa pegol group. The mean frequency of administration was 3.0 ± 1.5 and 2.2 ± 0.8 times/week in the octocog alfa and rurioctocog alfa pegol groups, respectively, demonstrating a lower frequency of dosing in the rurioctocog alfa pegol group compared to the octocog alfa group (*P* < 0.0001) (Table 2).

**Table 2:** Medical history and treatment

Variables	Octocog alfa (*N* = 48)	Rurioctocog alfa pegol (*N* = 81)	*P-*value
	Mean ± SD or N (%)	Mean ± SD or N (%)	
***Medical history***			
FVIII inhibitor history			
Yes (≥ 0.6 BU)	6(12.50)	5(6.17)	0.8737
No (< 0.6 BU)	42(87.50)	76(93.83)	
ITI history			0.4986
Yes	5(10.42)	5(6.17)	
No	43(89.58)	76(93.83)	
ITI Completion			
Yes	5(10.42)	5(6.17)	
ITI Result			-
Complete response	5(10.42)	5(6.17)	
Partial Response/Fail	-	-	
***Treatment information***			
FVIII treatment regimen at the time of PK test			0.2811
Prophylaxis	40(83.33)	72(88.89)	
On-demand	4(8.33)	2(2.47)	
Prophylaxis + On-demand	2(4.17)	6(7.41)	
Other	1(2.08)	-	
Unknown	1(2.08)	1(1.23)	
Dose, IU/kg	29.09 ± 4.68	28.81 ± 4.04	0.1842
Frequency, Times per week	2.98 ± 1.50	2.20 ± 0.80	** < 0.0001**

*ITI* Immune tolerance induction, *SD* Standard deviation

### PK profiles of octocog alfa and rurioctocog alfa pegol

The FVIII activity level was tested using one-stage assay for most patients, with the test method remaining unknown for 4% (5/129) of patients. The half-life range was 6.3–15.2 h for octocog alfa and 10.4–23.9 h for rurioctocog alfa pegol, revealing over twofold interpatient variations for the same factor concentrate. Similarly, the time to 1% exhibited a range of 27.0–88.0 h for octocog alfa and 63.0–150.0 h for rurioctocog alfa pegol. Compared with octocog alfa, rurioctocog alfa pegol demonstrated a longer half-life (154.1%) and a longer time to 1% (176.4%) (Table 3).

**Table 3:** PK profiles of octocog alfa and rurioctocog alfa pegol

	Octocog alfa (*N* = 48)	Rurioctocog alfa pegol (*N* = 81)	Difference (%)^a^	*P*-value
	Median (min, max)	Median (min, max)		
Half-life (h)	9.90 (6.30, 15.20)	15.30 (10.40, 23.90)	154.06	< 0.0001
Time to 1% (h)	53.00 (27.00, 88.00)	94.00 (63.00, 150.00)	176.37	< 0.0001
Clearance (dL/h/kg)	0.04 (0.02, 0.08)	0.02 (0.01, 0.04)	40.00	< 0.0001

^a^Difference (%) = (values of rurioctocog alfa pegol/octocog alfa)*100

### Association between PK and patient characteristics

Half-life and time to 1% exhibited significant relationships with age for octocog alfa (*P* < 0.0001 and *P* = 0.0002, respectively). For rurioctocog alfa pegol, these parameters were significantly related to vWF:Ag (*P* = 0.0002 and *P* < 0.0001, respectively). Clearance showed relationships with age (*P* < 0.0002) in the octocog alfa group, and in the rurioctocog alfa pegol group, it demonstrated associations with BMI (*P* = 0.0090) and vWF:Ag (*P* = 0.0052) (Table 4).

**Table 4:** Regression analysis of PK profile and patient variables

Variables	Parameter	Octocog alfa			Rurioctocog alfa pegol		
		n	Estimate	*P*-value	n	Estimate	*P*-value
Half-life (h)	Age	48	0.106	** < 0.0001**	81	0.013	0.6850
	BMI	42	0.100	0.1460	72	-0.053	0.5490
	vWF:Ag	6	0.045	0.1026	18	0.046	**0.0002**
Time to 1% (h)	Age	48	0.589	**0.0002**	81	0.144	0.4800
	BMI	42	0.567	0.2166	72	0.179	0.7580
	vWF:Ag	6	0.331	0.0528	18	0.311	** < 0.0001**
Clearance (dL/h/kg)	Age	48	-0.001	**0.0002**	81	0.001	0.9200
	BMI	42	-0.001	0.1310	72	-0.001	**0.0090**
	vWF:Ag	6	-0.0002	0.1859	18	0.0000	**0.0052**

*BMI* Body mass index, *vWF* Ag, von Willebrand factor antigen

Analysis of the PK profiles of octocog alfa and rurioctocog alfa pegol revealed that both FVIII concentrates exhibited a shorter half-life and time to 1% as well as higher clearance in individuals with blood type-O than in those with non-blood type-O. However, no differences were observed in the PK profiles of octocog alfa and rurioctocog alfa pegol after controlling for age classification or inhibitor history. Additionally, the clearance of rurioctocog alfa pegol varied across the different BMI classifications (Table 5).

**Table 5:** PK profile of each product according to age, blood type, inhibitor history, and BMI classification

Variables	Octocog alfa (*N* = 48)			Rurioctocog alfa pegol (*N* = 81)		
	n	Median (min, max)	*P*-value	n	Median (min, max)	*P*-value
**Age**						
Half-life (h)						
< 6 years	3	8.30 (6.30, 10.60)	0.0941	-	-	
≥ 6– < 12 years	5	9.21 (7.20, 9.50)		-	-	
≥ 12–18 years	7	9.70 (7.80, 11.30)		11	14.80 (12.10, 19.10)	0.6807
≥ 18 years	33	10.30 (6.40, 15.20)		70	15.40 (10.40, 23.90)	
Time to 1% (h)						
< 6 years	3	45.00 (34.00, 58.00)	0.4220	-	-	
≥ 6– < 12 years	5	51.00 (38.00, 54.00)		-	-	
≥ 12– < 18 years	7	52.00 (38.00, 62.00)		11	88.00 (67.00, 108.00)	0.3315
≥ 18 years	33	54.00 (27.00, 88.00)		70	95.00 (63.00, 150.00)	
Clearance (dL/h/kg)						
< 6 years	3	0.05 (0.04, 0.07)	0.0829	-	-	
≥ 6– < 12 years	5	0.05 (0.05, 0.06)		-	-	
≥ 12– < 18 years	7	0.05 (0.04, 0.06)		11	0.02 (0.02, 0.03)	0.1015
≥ 18 years	33	0.04 (0.02, 0.08)		70	0.02 (0.01, 0.04)	
**Blood type**						
Half-life (h)						
O	15	8.30 (6.40, 10.60)	** < 0.0001**	12	12.65 (10.40, 18.00)	** < 0.0001**
Non-O	26	10.6 (6.30, 15.20)		53	15.70 (10.40, 23.90)	
Time to 1% (h)						
O	15	42.00 (27.00, 56.00)	** < 0.0001**	12	75.00 (63.00, 112.00)	** < 0.0001**
Non-O	26	57.00 (34.00, 88.00)		53	96.00 (66.00, 150.00)	
Clearance (dL/h/kg)						
O	15	0.06 (0.04, 0.08)	** < 0.0001**	12	0.03 (0.02, 0.04)	** < 0.0001**
Non-O	26	0.04 (0.02, 0.07)		53	0.02 (0.01, 0.02)	
**Inhibitor history**						
Half-life (h)						
Yes (≥ 0.6 BU)	6	8.50 (6.30, 13.90)	0.2082	5	15.40 (10.40, 15.60)	0.1469
No (< 0.6 BU)	42	9.95 (6.40, 15.20)		76	15.30 (10.40, 23.90)	
Time to 1% (h)						
Yes (≥ 0.6 BU)	6	41.00 (34.00, 77.00)	0.2355	5	94.00 (66.00, 95.00)	0.2025
No (< 0.6 BU)	42	53.00 (27.00, 88.00)		76	93.50 (63.00, 150.00)	
Clearance (dL/h/kg)						
Yes (≥ 0.6 BU)	6	0.05 (0.03, 0.07)	0.1423	5	0.02 (0.02, 0.02)	0.6368
No (< 0.6 BU)	42	0.04 (0.02, 0.08)		76	0.02 (0.01, 0.04)	
**BMI (kg/m**^**2**^**)**						
Half-life (h)						
< 18.5	7	8.40 (6.30, 11.70)	0.2139	2	14.65 (10.50, 22.50)	0.8583
18.5–22.9	13	9.90 (7.80, 11.90)		21	15.40 (11.90, 19.20)	
23.0–24.9	5	10.60 (8.90, 15.20)		18	14.45 (10.40, 23.90)	
≥ 25.0	17	10.30 (6.40, 13.40)		31	15.40 (10.40, 23.90)	
Time to 1% (h)						
< 18.5	7	48.00 (34.00, 64.00)	0.2882	2	84.00 (63.00, 137.00)	0.5681
18.5–22.9	13	53.00 (39.00, 64.00)		21	91.00 (70.00, 117.00)	
23.0– 24.9	5	55.00 (46.00, 88.00)		18	88.50 (64.00, 150.00)	
≥ 25.0	17	54.00 (27.00, 79.00)		31	95.00 (64.00, 150.00)	
Clearance (dL/h/kg)						
< 18.5	7	0.05 (0.04, 0.07)	0.3039	2	0.02 (0.02, 0.03)	**0.0029**
18.5–22.9	13	0.05 (0.03, 0.06)		21	0.02 (0.02, 0.04)	
23.0–24.9	5	0.04 (0.02, 0.05)		18	0.02 (0.01, 0.03)	
≥ 25.0	17	0.04 (0.03, 0.08)		31	0.02 (0.01, 0.03)	

*BMI* Body mass index

### Dose and frequency variations according to PK

**Fig. 1:**
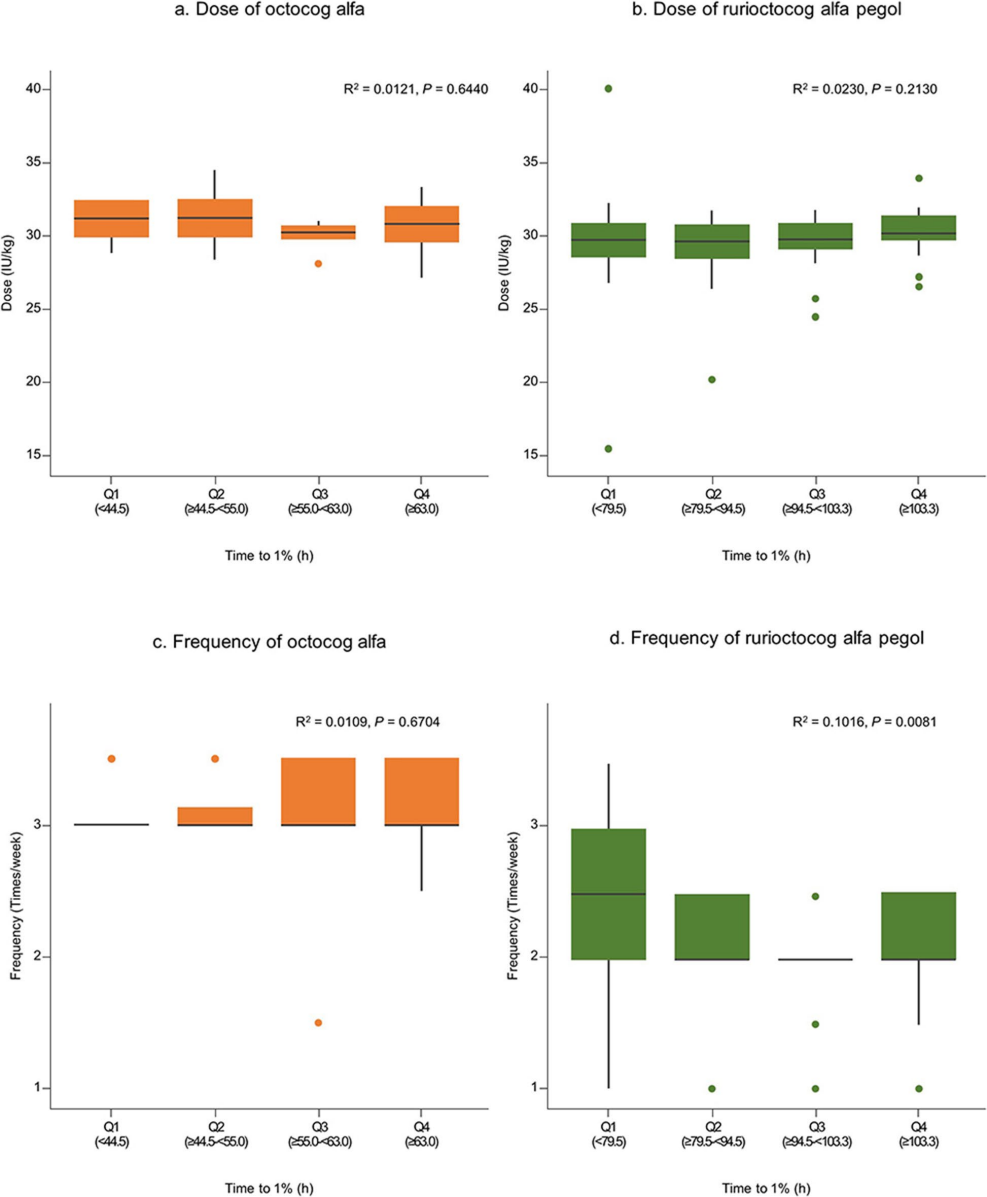
Distribution of dose and frequency by quartiles of time to 1%. **A** Dose (IU/kg) of octocog alfa; (**B**) dose of rurioctocog alfa pegol; (**C**) frequency (injections per week) of octocog alfa; and (**D**) frequency of rurioctocog alfa pegol. The box in the plot represents the interquartile range (IQR), and the points extending from the box represent values that are 1.5 times greater than the IQR from the median value. *R*^2^ was calculated using a simple linear regression

The distribution of the dose and frequency of octocog alfa and rurioctocog alfa pegol in severe cases is categorized into quartiles based on the time to 1% for each product. The dose and frequency did not differ for either product except for the frequency of rurioctocog alfa pegol. However, the coefficient of determination was 0.1016 (Fig. 1).

**Fig. 2:**
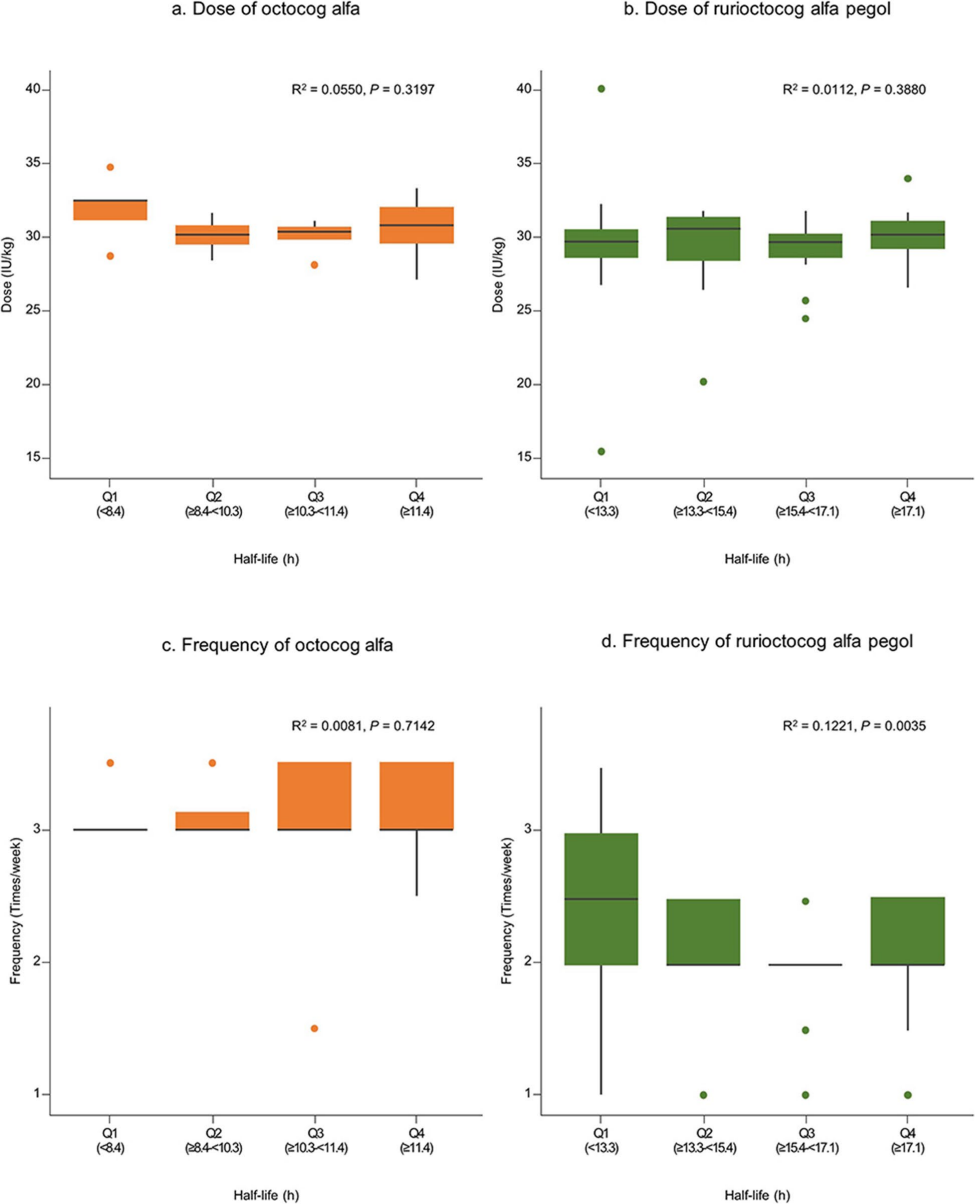
Distribution of dose and frequency by quartiles of half-life. **A** Dose (IU/kg) of octocog alfa; (**B**) dose of rurioctocog alfa pegol; (**C**) frequency (injections per week) of octocog alfa; and (**D**) frequency of rurioctocog alfa pegol. The box in the plot represents the interquartile range (IQR), and the points extending from the box represent values that are 1.5 times greater than the IQR from the median value. R^2^ was calculated using a simple linear regression

A similar result was obtained for half-life (Fig. 2).

## Discussion

Interpatient variations in the PK of FVIII have been reported in individuals with hemophilia. Based on these studies, various international organizations, such as the World Federation of Hemophilia (WFH), the United Kingdom Haemophilia Centre Doctors’ Organization (UKHCDO), and the National Bleeding Disorders Foundation (NBDF), recommend personalized prophylaxis that considers the unique PK profile of each patient [1, 8, 9]. Consistent with this trend, this study established a basis for the implementation of PK-based personalized prophylaxis in Korea.

Consistent with our findings, several studies have reported wide variations in PK profiles. A simulation study, utilizing data from prospective clinical trials with octocog alfa, demonstrated a significant difference in time to 1% between patients in the 5th and 95th percentiles of half-lives, with a gap of 57.1 h (range: 46.4–103.5 h) [3]. Another Canadian study presented a half-life range of 11.0–23.6 h in adolescent patients for rurioctocog alfa pegol [10]. Similarly, the reported half-life values for a recombinant FVIII among Chinese pediatric patients showed a range of 7.1–22.6 h [11]. However, real-world PK data for Asian patients, particularly for FVIII concentrates with extended half-lives, remains limited.

In addition, although the objective of this real-world study in Korean patients was not to directly compare the PK profiles of rurioctocog alfa pegol and octocog alfa, the half-life and time to 1% for rurioctocog alfa pegol were more than 1.5-fold longer than those of octocog alfa, whereas the clearance of rurioctocog alfa pegol was approximately 0.4 times-that of octocog alfa.

A linear relationship was observed between age and the PK of octocog alfa, which was characterized by an increase in half-life and time to 1%, along with a decrease in clearance. This finding aligns with a study conducted by Björkman, who also reported linear positive and negative correlations with the half-life and clearance of FVIII, respectively, based on age [12]. However, in the case of rurioctocog alfa pegol, the present study did not observe statistically significant relationships between the PK parameters and age. This may be attributed to the exclusion of children under 12 years of age from the study owing to limitations in the PK simulation using myPKFiT.

Recovery of FVIII is increased in patients with a higher BMI because the increase in body weight is primarily due to increased adipose tissue, which contains less vascular space than lean mass. Consequently, obese individuals are likely to have a higher circulating plasma FVIII level [13]. This study did not assess the recovery of FVIII; however, BMI showed a linear relationship with the clearance of rurioctocog alfa pegol. This result is consistent with the reported effects of BMI on clearance, as clinical trials have reported a negative correlation between BMI and clearance [14, 15]. However, in the case of octocog alfa, the study did not find an association between BMI and clearance, which may be attributed to the inclusion of data from pediatric patients who have a larger volume of distribution than adults because of greater extracellular and total body water space. Additionally, metabolic rate, which varies with age, may also have an effect. In the BMI group analysis, the Korean BMI classification was used, which differs from that of the World Health Organization (Table 6), and this distinction must be considered [16, 17].

**Table 6:** BMI classifications (adults)

[unit: kg/m^2^]	Underweight	Normal	Overweight	Obesity
Korea [16]	< 18.5	18.5–22.9	23.0–24.9	≥ 25.0
WHO [17]	< 18.5	18.5–24.9	25.0–29.9	≥ 30.0

This study also found a linear relationship between vWF:Ag and half-life, time to 1%, and clearance in rurioctocog alfa pegol, which is consistent with previous reports [18–20]. The relationship between vWF:Ag and blood type is known [21], with non-blood type-O individuals having higher vWF:Ag levels than blood type-O individuals in patients with hemophilia [22]. The PK analysis by blood type (O vs. non-blood type-O) revealed that the half-life and time to 1% were reduced in blood type-O and increased in non-blood type-O patients, whereas the opposite result was observed for the clearance of both products. Similar results have been reported in other studies [14, 22, 23]. However, because of the small sample size (octocog alfa, 6/48; rurioctocog alfa pegol, 18/81), this study acknowledges that vWF:Ag, measured at an interval close to the PK test, may have failed to demonstrate a statistical correlation with the PK profile of octocog alfa.

The dose and frequency by the PK parameters were analyzed in severe cases, which revealed no significant differences in patients stratified by quartiles of time to 1% and half-life. However, the frequency of rurioctocog alfa pegol showed a statistically significant difference based on time to 1% and half-life. Although statistically significant, the impact may not be meaningful because of the relatively small coefficients of determination (*R*^2^ = 0.1016 for time to 1% and *R*^2^ = 0.1221 for half-life). In this study, the mean dose and frequency of octocog alfa were 29.1 ± 4.7 IU/kg and 3.0 ± 1.5 times/week, respectively, while for rurioctocog alfa pegol, the mean dose and frequency were 28.8 ± 4.0 IU/kg and 2.2 ± 0.8 times/week, respectively. These values are very close to the upper limit of the reimbursement guidelines for patients with severe hemophilia A in Korea (up to 30 IU/kg, 3 times/week for standard half-life FVIII concentrates, such as octocog alfa, and 30 IU/kg, 2 times/week for extended half-life FVIII concentrates, such as rurioctocog alfa pegol) [7].

Similar to other studies, this study revealed that the time to 1% and half-life of each product varied by more than 2–3 times among Korean patients. Personalized prophylaxis, which involves adjusting the dose and frequency based on individual PK profiles, is recommended for the optimal treatment of hemophilia. However, despite the observed PK variations among the patients, the prophylaxis dose and frequency of administration were close to the upper limit of reimbursement, regardless of the individual’s time to 1% or half-life. The limited flexibility to increase the dose or frequency is a cause for concern, particularly because PK tests are preferentially performed in patients with insufficient treatment outcomes under the current prophylaxis regimen.

A recent update to the reimbursement guidelines for FVIII concentrates permits increased doses in cases where the trough FVIII level is below 1% at the time points of 48 h and 72 h after the administration of standard and extended half-life FVIII concentrates, respectively [24, 25]. This change is significant because it acknowledges the variations in individual PK. However, the 1% target trough FVIII level is considerably lower than the 3–5% recommended by the WFH. This discrepancy implies that patients still face a risk of bleeding [1].

A few years ago, the WFH revised the definition of prophylaxis, emphasizing its purpose as a treatment that allows patients with hemophilia to perform physical and social activities akin to non-patients, prompting healthy and active lives [1]. Therefore, there is a need for practices in Korea that reflect the interpatient variation observed in hemophilia. In addition to the PK-based personalized prophylactic regimen for patients with hemophilia A, considering individual and situation-specific dosing schedules to maintain an appropriate factor level according to the time of physical and social activity is important. The use of a patient application with real-time FVIII level estimation and patient education to properly utilize this information are also recommended.

To the best of our knowledge, this is the only real-world study analyzing the PK of FVIII in Korean patients, including a substantial number of patients receiving FVIII with an extended half-life. However, this study had some limitations; for example, children under 12 years of age could not be enrolled in the rurioctocog alfa pegol study because PK simulation with myPKFiT cannot be performed in this age group. Missing data, particularly concerning the vWF:Ag level, were attributed to the retrospective nature of this study. Additionally, the number of patients with a history of inhibitor titers of 0.6 BU or higher was small, limiting the analysis of the correlation with the PK profile. Future studies should focus on regimen changes based on individual PK profiles and treatment outcomes.

This study underscores the significant interpatient variation in the PK of FVIII concentrates in Korean patients with hemophilia A. To optimize the effectiveness of prophylactic regimens and improve the overall treatment outcomes, a personalized approach must be implemented and tailored according to an individual patient’s PK profile. Moreover, treatment strategies should target the prevention of bleeding and enable healthy and active lifestyles in patients with hemophilia.

## Data Availability

The datasets, including the redacted study protocol, redacted statistical analysis plan, and individual participant’s data supporting the results reported in this article, will be made available within three months from initial request to researchers who provide a methodologically sound proposal. The data will be provided after its de-identification, in compliance with applicable privacy laws, data protection and requirements for consent and anonymization.
